# Effects of dual-task resistance exercise on cognition, mood, depression, functional fitness, and activities of daily living in older adults with cognitive impairment: a single-blinded, randomized controlled trial

**DOI:** 10.1186/s12877-024-04942-1

**Published:** 2024-04-24

**Authors:** Ji-Eun Baek, Sang-Jun Hyeon, May Kim, Hwi-young Cho, Suk-Chan Hahm

**Affiliations:** 1 Department of Physical Therapy, College of Health Science, Gachon University, Incheon, Republic of Korea 191 Hambangmoe-ro, Yeonsu-gu Incheon; 2 Graduate School of Integrative Medicine, CHA University, 120 Haeryong-ro, Pocheon-si, 11160 Kyonggi-do Republic of Korea; 3 Department of Physical Education, College of Education, Korea University, Seoul, Republic of Korea

**Keywords:** Dual-task resistance exercise, Resistant exercise, Cognitive impairment, Cognition, Mood, Functional fitness, Activities of daily living

## Abstract

**Background:**

Regular exercise is emphasized for the improvement of functional capacity and independence of older adults. This study aimed to compare the effects of a dual-task resistance exercise program and resistance exercise on cognition, mood, depression, physical function, and activities of daily living (ADL) in older adults with cognitive impairment.

**Methods:**

A total of 44 older adults participated in the study. Participants were randomly allocated to an experimental group (*n* = 22) performing a dual-task resistance exercise program for cognitive function improvement and a control group (*n* = 22) performing a resistance exercise program. Both groups performed the exercise for 40 min per session, three times a week, for 6 weeks (18 sessions). Cognition, mood, depression, functional fitness, and ADL were quantified before and after the intervention using the Mini-Mental State Examination (MMSE), profile of mood states (POMS), geriatric depression scale (GDS), senior fitness test (SFT), and Korean version of ADL, respectively.

**Results:**

There was a significant time and group interaction on the MMSE (*p* = 0.044). There were no significant time and group interactions in the POMS, GDS, SFT, or ADL. Cognitive function (*p* < 0.001), mood (*p* < 0.001), depression (*p* < 0.001), functional fitness (*p* < 0.001), and ADL (*p* < 0.001) significantly improved after dual-task resistance exercise, and cognitive function (*p* < 0.001), mood (*p* < 0.001), depression (*p* < 0.001), functional fitness (*p* < 0.001), and ADL (*p* < 0.001) significantly improved after resistance exercise.

**Conclusions:**

Dual-task resistance exercise is more effective than resistance exercise in improving cognitive function in older adults with cognitive impairment. Both dual-task resistance exercise and resistance exercise improves mood, depression, functional fitness, and ADL after the intervention. We propose using dual-task resistance exercises for cognitive and physical health management in the older adults with cognitive impairment.

**Trial registration:**

This study was registered with the Clinical Research Information Service (WHO International Clinical Trials Registry Platform) (Registration ID, KCT0005389; Registration date, 09/09/2020).

## Introduction

 The global aging phenomenon, an indispensable topic in modern society, has sharply increased interest in the lives of the older adult population. Therefore, unlike in ancient times when geriatric diseases were considered natural phenomena, maintaining and improving a healthy body has become a significant social concern [1]. According to a report released by the United Nations in 2020, the number of people aged 65 and over globally is estimated to be 728 million and is expected to reach 1.5 billion by 2050 [2]. In addition, the U.S. Centers for Disease Control and Prevention (2019) reported that 11.7% of adults over the age of 65 suffer from cognitive impairment [3]. Aging is associated with cognitive impairment and contributes to a decline in physical function [4, 5]. Decline in cognitive and physical function can reduce activities of daily life (ADL), leading to feelings of loneliness, alienation, and psychological atrophy, leading to an increase in depression in older adults [6–8]. Decreased physical functions may increase the risk and fear of falls, the incidence of geriatric diseases, and lead to a decrease in self-confidence in performing ADL [9, 10].

Physical exercise is considered an essential healthcare option for older people [11]. Regular exercise can have positive cognitive and physical effects, such as delaying aging-induced neurodegeneration and reducing the risk of falls and depression in older adults [12, 13]. Therefore, various studies are being conducted to improve older adults’ cognitive and physical functions. Resistance exercise is mainly used to prevent deterioration of physical functions due to aging [14]. Various literatures have also reported that resistance exercise helps to improve cognitive function by causing structural and functional changes in the brain [15–18].

Dual-task exercise program that combines cognitive training and physical activity programs for older adults with mild cognitive impairment has been reported [19]. Recent studies have also reported that dual-task training improved balance and gait function in patients with stroke [20] and spatio-temporal parameters of gait in older women with dementia [21]. A previous study has reported that exercises that include dual-task could be the key of therapeutic success to slow down the progression of dementia, but risk of falls during dual task performance compared to single task performance was presented in patients with dementia [22]. A recent systematic review suggested a further study on the capability of cognitive-motor dual-tasks related to falls in patients with stroke [23].

Recent research highlights the effectiveness of resistance exercise interventions in improving physical functionality, mood, and alleviating depression among older adults [14–18]. Additionally, previous studies have reported that exercises that applies dual tasks has demonstrated potential in enhancing cognitive capabilities [24–28]. Based on the findings of these previous studies, it is necessary to perform resistance exercise for the comprehensive management of cognition, emotion, depression, and physical health in older adults. Additionally, adopting a dual-task-based approach is crucial as it holds potential for enhancing cognitive function. The appropriate exercise program for managing older adults’ health is dual-task resistance exercise, which can deliver all the benefits of resistance exercise while additionally enhancing cognitive function.

Although there are various reports, controversy exists about dual-task interventions for the rehabilitation of patients with cognitive impairment and the efficacy of exercise programs to improve mood, depression, physical function, and ADL, along with improving cognitive function in older adults with cognitive impairment, is still insufficient. A recent systematic review and meta-analysis has reported positive effects of dual-task exercise on cognition and motor function in older adults with cognitive impairment, but several studies included in the systematic review compared experimental groups with control groups such as health education and placebo activities, not active comparators [29]. Studies in which the risk of selection bias existed were also included. Therefore, it is necessary to prepare evidence through methodologically appropriately designed studies to minimize bias.

This study aimed to examine the effects of an exercise program to improve cognition, mood, depression, physical function, and ADL in older adults with cognitive impairment. The experimental group performed a dual-task resistance exercise program aimed at improving cognitive function, and the control group performed a resistance exercise program. We hypothesized that dual-task resistance exercise would be more effective in improving cognition compared to resistance exercise. Additionally, we hypothesized that dual-task resistance exercise would have similar effects on mood, depression, physical function, and activities of daily living (ADL) as resistance exercise.

## Methods

### Design and ethic

This study was designed as a single-blinded, randomized controlled trial. This study was approved by the CHA University Institutional Review Board on August 14, 2020 (No.1044308-202006-HR-019-02). This study was registered with the Clinical Research Information Service (WHO International Clinical Trials Registry Platform) on September 9, 2020 (study identifier, KCT0005389). Before participating in the study, participants were informed about the research procedure, and informed consent was obtained from all participants involved.

### Participants

Among those wishing to participate, those who satisfied the following conditions were selected as participants [30, 31]; (1) those who were 65 years of age or older, (2) those with The Mini-Mental State Examination-Korean (MMSE-K) score of 23 or less, (3) those who could walk without a walking aid, and (4) those who could communicate in Korean and follow the instructions of the research team. The exclusion criteria were as follows: (1) history of stroke with movement impairment, (2) history of traumatic brain injury with a movement disorder, and (3) neurological or mental comorbidity other than cognitive impairment.

### Sample size

To calculate the sample size, G*Power 3.1.9.7 (Universität Kiel, Kiel, Germany) was used, with an effect size of 0.25 (medium effect size) [32], ANOVA (repeated measures, within-between interaction), and two repeated measures. Thirty-four participants were required to achieve 80% power at an α level of 0.05. Based on these values, 43 participants were required, with a 20% dropout rate.

### Intervention

**Fig. 1:**
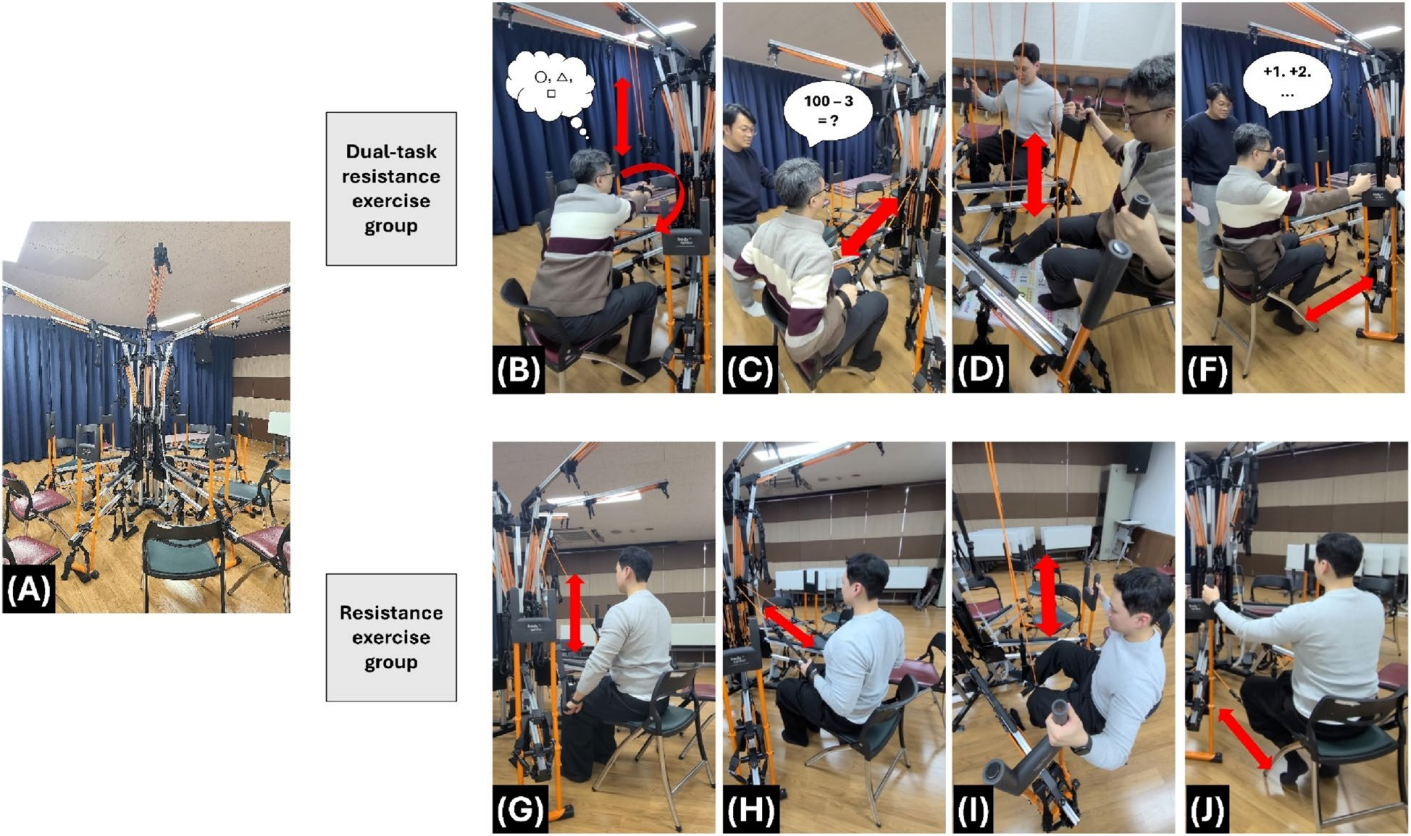
Dual-task resistance exercise and resistance exercise. **A** Body Spider, **B**-**F** Dual-task resistance exercise, **G**-**J** Resistance exercise

 Each group performed an exercise using a body spider (KOOPERA, Germany) for 40 min per session, three times a week, for 6 weeks (18 sessions) [33]. A body spider is an exercise device that uses the elasticity of a rubber rope, and both concentric and eccentric contractions are possible. It comprises a three-stage frame of the upper, middle, and lower parts; therefore, it is capable of whole-body exercise in various directions. Body spider was also used to confirm the effects of exercise in older adults [34]. The experimental group performed a dual-task resistance exercise program to improve cognitive function, and the control group performed a resistance exercise program for the same period using the same equipment (Table 1; Fig. 1). The individuals included in Fig. 1 are members of our research team, and we obtained written consent from them for publication.In both groups, warm-up exercise was performed for 10 min before main exercise and cool-down exercise was performed for 10 min after main exercise. The experimental group performed dual tasks such as writing names, drawing pictures, and subtracting numbers while performing the same resistance exercise applied to the control group. The control group performed resistance exercises for 20 min. At this time, the strength of the resistance exercise was measured at 1RM, and 3 sets were performed with approximately 10 reps per set, with a maximum of 12RM.

**Table 1:** Dual-task resistance exercise and resistance exercise

Categories	Dual-task resistance exercise	Resistance exercise
Warm-up exercise (10 min)	Clapping, shoulders, ankles, and toes stretching, knees pulling to chest, chair stand up.	
Main exercise (20 min)	(B) While sitting on a chair, pull the pulley from top to bottom to write names and numbers and draw shapes.	(F) While sitting on a chair, pull the pulley from top to bottom.
	(C) While sitting on a chair, pull the pulley parallel to the ground with your arm and repeat the mental arithmetic by subtracting 3 from 100.	(G) While sitting on a chair, pull the pulley parallel to the ground with your arm.
	(D) While sitting on a chair, hook a pulley to your feet and move your feet in line with the numeric footrests installed on the floor.	(H) While sitting on a chair, hook a pulley to your feet and flex your hip joint.
	(E) While sitting on a chair, hang a pulley on your ankle, bend your knees to pull the pulley parallel to the ground, and repeat mental arithmetic adding 1 to 2.	(I) While sitting on a chair, hang a pulley on your ankle, bend your knees to pull the pulley parallel to the ground.
Cool-down exercise (10 min)	Neck, shoulder, and trunk rotation, upper and lower extremities brushing, breathing.	

### Outcome measures

#### Primary variable (cognitive function)

The MMSE-K was used to investigate changes in cognitive function. The MMSE-K consists of time orientation (five points), place orientation (five points), memory registration (three points), memory recall (three points), attention and calculation ability (five points), language function (seven points), and comprehension (two points). It consists of seven sub-items of judgment ability (two points), with a total score of 30 points. When the MMSE-K score exceeds 24, it is classified as definitively normal, and when it is under 24, it is judged that the cognitive function has deteriorated. This tool has excellent validity (0.93) [35].

#### Secondary variables (Mood state, Depression state, functional fitness, activities of daily living)

The Korean version of the Profile of Mood States (K-POMS) was used to assess participants’ mood states. The mood state profile has a total of 30 items and consists of six sub-domains: tension, depression, anger, vigor, fatigue, and confusion. Responses were categorized using a 5-point Likert scale with 0, 1, 2, 3, and 4 points for ‘not at all,’ ‘a little,’ ‘moderately,’ ‘quite a lot,’ and ‘extremely,’ respectively. The total mood disturbance is the sum of the scores of all five negative indicators (tension, depression, anger, fatigue, and confusion) among the subcategories minus the positive indicator (vigor). A higher total score for mood state is interpreted as a lower mood state for the participant. The reliability coefficient is 0.93 [36].

The Korean geriatric depression scale (GDS-K) was used to assess older adults with the symptoms of depression. This measurement method is a nutrient scale in which the participant responds with yes/no, and 18 or more out of 30 items indicate depression. The Cronbach’s alpha coefficient of the GDS-K is 0.90 [37].

Functional fitness was evaluated using the Senior Fitness Test (SFT) compiled by Rikli and Jones in 1999 [38]. Among the sub-items of the senior fitness test, a hand-held dynamometer instead of the 30-sec arm curl was used to evaluate upper extremity strength, a 30-sec chair stand to evaluate the remaining lower extremity strength, a 2-min step walk to evaluate endurance, 244 cm up and go to evaluate agility, back scratch to evaluate upper extremity flexibility, and chair sit and reach to evaluate lower extremity flexibility. Additionally, a one-leg standing test was used to evaluate static balance [39]. These assessments are also useful to investigate the risk of falls in older adults [40–42].

The Korean ADL (K-ADL) scale was used to quantify the participants’ ADL. The tool completed seven sub-items (dressing, washing face, bathing, eating, moving, using the toilet, and toileting) on a 3-point scale: (1) able to do alone without assistance, (2) need partial help, and (3) totally need help. It was structured to respond on a scale. The Cronbach’s alpha of the K-ADL was 0.93 [43].

### Procedure

In this study, A recruitment notice was posted at a senior day care center located in Goyang, South Korea. Participants who agreed to participate and met the inclusion criteria were randomly assigned to experimental and control groups using permuted block randomization (block size 4). Allocation concealment was performed by placing sequentially numbered, opaque, and sealed envelopes. Participants were randomly allocated to an experimental group that performed a dual-task resistance exercise program for cognitive function improvement and a control group that performed a resistance exercise program. All assessments and interventions were conducted in the senior day care center.

Interventions were conducted for 40 min per session, three times a week, 18 times for 6 weeks, from October 13 to November 21, 2020 (18 sessions). All interventions were performed under the supervision of two physical therapists with 3 or more years of clinical experience. Five (or four) older adults and one physical therapist were a team. Both the experimental group and the control group were composed of 5 teams. Participants performed exercises using body spiders following the instructions of the physical therapist. Except for the chair stand up of the warm-up exercise, all exercises were performed in a sitting position. A physical therapist in each group instructed each group’s exercise until all interventions were completed.

The exercise program conducted in this study was directly supervised by a physical therapist, with the duration set at a level that ensured participants with cognitive impairment did not experience undue discomfort or burden. During all interventions and assessments, researchers and safety personnel stood right next to the participants in case anything went wrong with them. Participants were informed that they were free to withdraw from the program if they felt burdened during the study period.

When assessing cognitive function, mood state, depressive state, functional fitness, and activities of daily living, group information was not provided to the assessor. Before the intervention, the participants were asked to complete the questionnaire, and participants who had difficulty reading texts were allowed to complete the questionnaire as the researcher read it. After 18 interventions, the same variables were assessed in the same way.

### Statistical analysis

All data were analyzed using SPSS 25.0 (SPSS Inc., Chicago, IL, USA). For comparison between groups on the general characteristics of participants, the chi-square test was used for categorical variables, and the independent t-test was used for continuous variables. A mixed analysis of variance was performed to analyze the time and group interaction for each dependent variable. A paired t-test was performed for the average difference between two time points in each group, and the significance level was corrected using Bonferroni correction. The statistical significance level was set at α = 0.05.

## Results

### General characteristics of participants

**Fig. 2:**
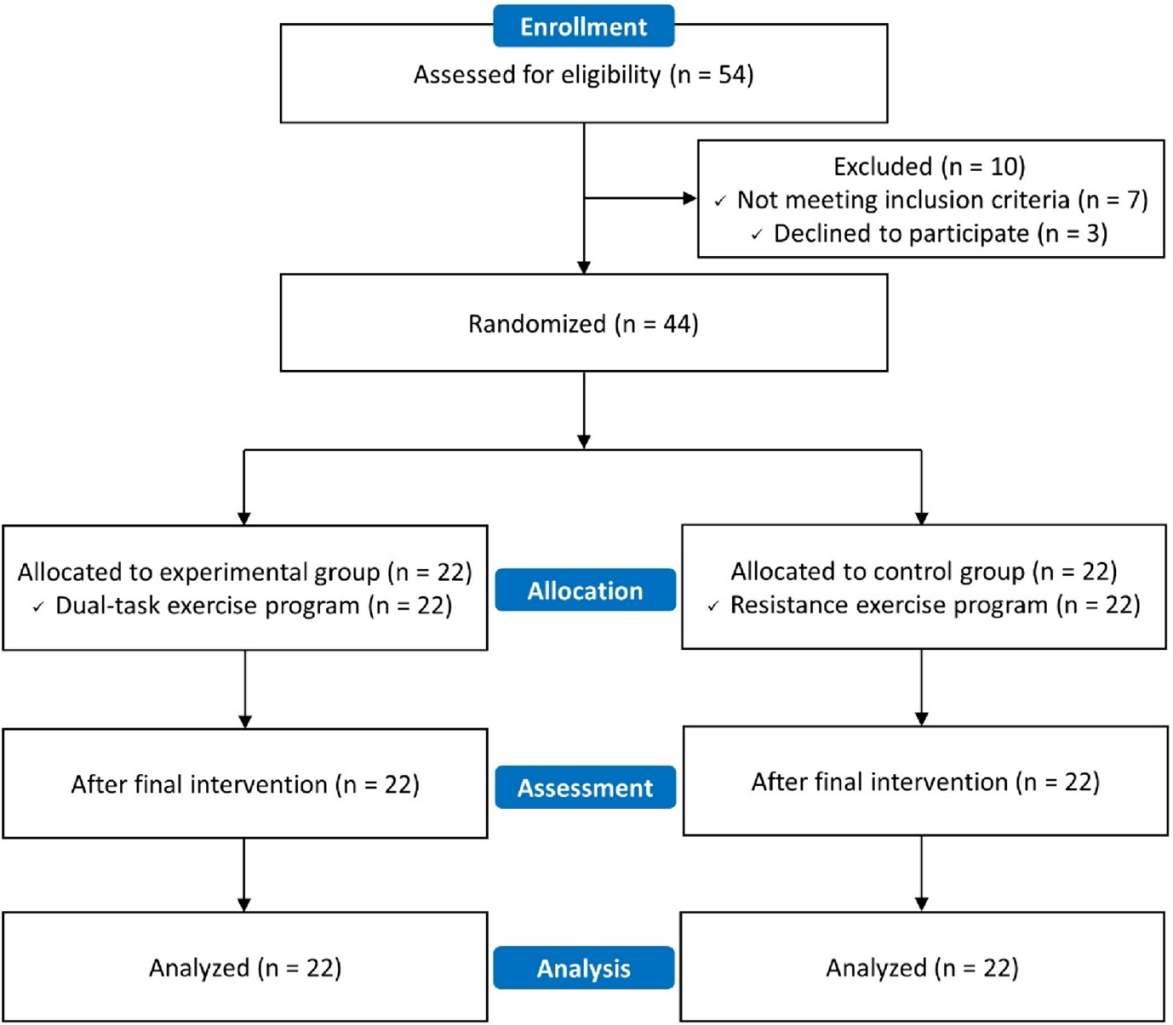
Flow diagram of participants

 Of the 54 recruits, three declined to participate and seven met the exclusion criteria. A total of 44 people participated in the experiment, and the experiment was conducted by randomly dividing the participants into 2 groups. No person was excluded from the assessment or analysis (Fig. 2).

The general characteristics of the participants are presented (Table 2). Among the 44 participants, 15 (34.1%) males and 29 (65.9%) females were divided into 22 in the experimental group and 22 in the control group. Regarding sex, 7 males and 15 females were in the exercise group, and 8 males and 14 females were in the control group. There was no statistically significant difference between the experimental and control groups in sex ratio, mean age, height, weight, and body mass index. Cognitive function, mood state, depression state, functional fitness, activities of daily living showed no significant differences of pre-values between two groups.

**Table 2:** General characteristics of participants at the baseline

	Dual-task resistance exercise (*n* = 22)	Resistance exercise (*n* = 22)	* p*-value
Sex (n)			
Male	7	8	1.000
Female	15	14	
Age (year)	82.40 ± 4.46	81.04 ± 4.93	0.342
Height (cm)	154.94 ± 8.13	155.30 ± 7.34	0.877
Weight (kg)	56.82 ± 11.96	56.87 ± 12.09	0.990
BMI (kg/m^2^)	23.64 ± 4.20	23.42 ± 3.85	0.860
MMSE (score)	16.04 ± 2.23	16.09 ± 1.97	0.943
POMS (score)	72.18 ± 3.96	72.41 ± 3.72	0.845
GDS (score)	20.23 ± 1.23	20.86 ± 1.55	0.139
Senior fitness test			
Back scratch (cm)	-10.41 ± 11.51	-5.23 ± 9.03	0.104
Chair sit & reach (cm)	-8.86 ± 9.76	-5.23 ± 10.85	0.249
244 cm up & go (sec)	15.85 ± 4.83	18.49 ± 5.37	0.093
2-min step walk (number)	26.91 ± 5.33	27.18 ± 4.60	0.857
30-sec chair stand (number)	7.41 ± 1.92	7.77 ± 2.16	0.558
Grip strength – Lt. (kg)	15.70 ± 7.34	17.42 ± 7.97	0.460
Grip strength – Rt. (kg)	16.97 ± 6.86	18.45 ± 8.58	0.530
One leg standing (sec)	3.10 ± 0.83	2.70 ± 0.58	0.074
ADL (score)	13.41 ± 1.18	13.82 ± 1.33	0.287

Data are expressed as mean ± SD or number of participants

*BMI *Body mass index, *MMSE *Mini-mental state examination, *POMS *Profile of mood states, *GDS *Geriatric depression scale, *ADL *Activities daily of living

#### Cognitive function

**Fig. 3:**
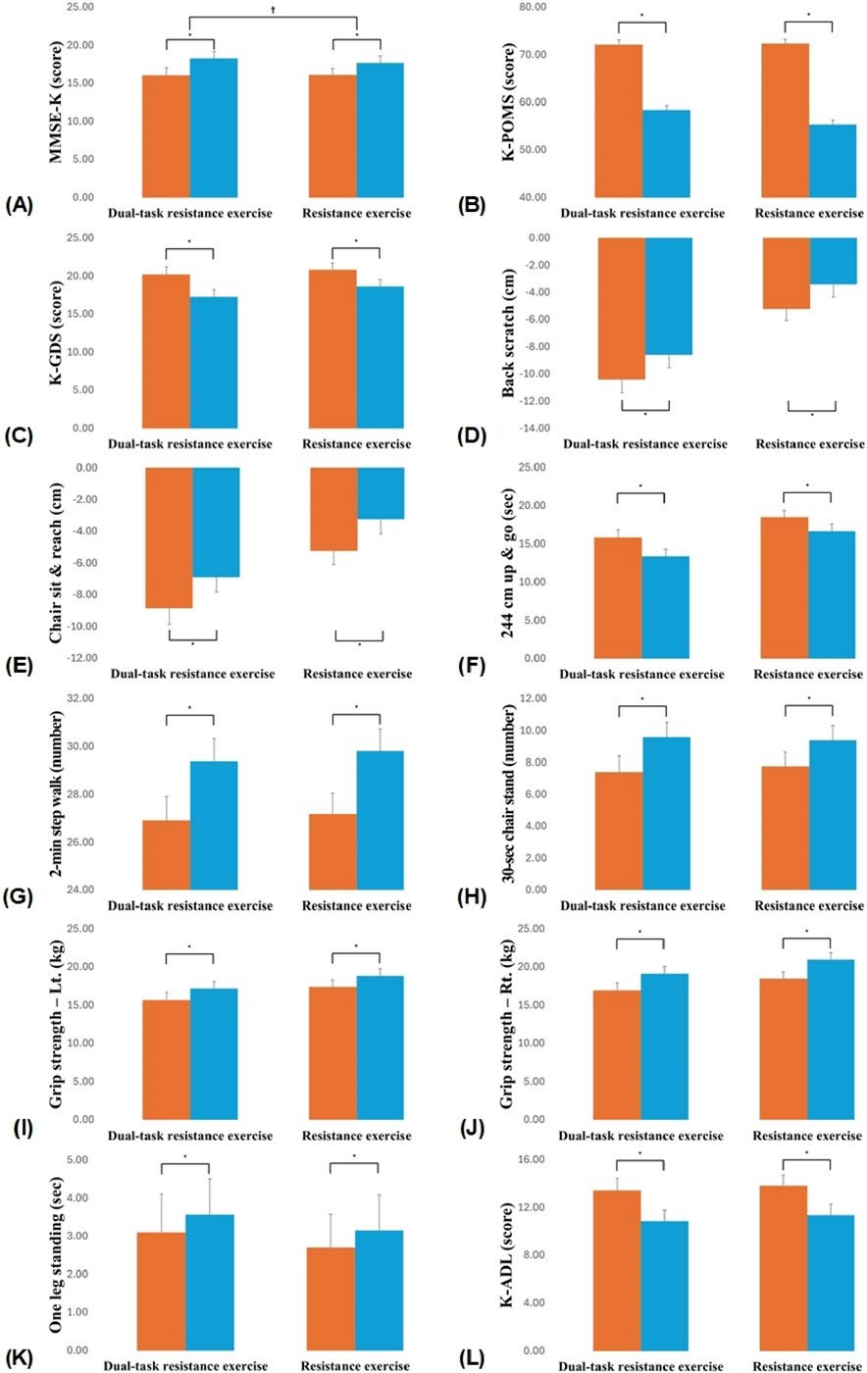
Comparisons of changes for each variable. Observed changes in each variable from pre- to post-intervention. **A** MMSE-K, **B** K-POMS, **C** K-GDS, **D** Back scratch, **E** Chair sit and reach, **F** 244 cm up and go, **G** 2-min step walk, **H** 30-sec chair stand, **I** Grip strength – Lt., **J** Grip strength – Rt., **K** One leg standing, **L** K-ADL. Orange-colored bars represent pre-intervention, while Blue-colored bars represent post-intervention. Bar length and error bar expressed mean and standard deviation, respectively. *Significant difference in within-group; ^†^Significant time and group interaction. MMSE-K, mini-mental state examination-KOREA; K-POMS, Korean-profile of mood states; K-GDS, Korean-geriatric depression scale, Lt., Light; Rt., Right; K-ADL, Activities daily of living

 Table 3 shows the changes in cognitive function over time between the experimental and control groups (Table 3) (Fig. 3). There was significant time and group interaction for cognitive function (F = 4.333, *df* = 1, *p* = 0.044). The cognitive scores of the experimental group significantly increased after the intervention (*p* < 0.001). The control group also showed a significant increase in cognitive function post-intervention (*p* < 0.001).

**Table 3:** Cognitive function, mood, and depression

	Dual-task resistance exercise			Resistance exercise			Between-group difference in change scores^a^	*p*-value (T×G)	Partial η^2^
	pre	post	Within-group change score^a^	pre	post	Within-group change score^†^			
MMSE (score)	16.05 ± 2.24	18.27 ± 2.12	2.23 (1.70, 2.76)	16.09 ± 1.97	17.68 ± 2.10	1.59 (1.24, 1.94)	0.64 (0.02, 1.25)	0.044^*^	0.094
POMS (score)	72.18 ± 3.96	58.41 ± 5.16	-13.77 (-16.67, -10.88)	72.41 ± 3.73	55.36 ± 5.21	-17.05 (-19.01, -15.08)	-3.27 (-6.67, 0.12)	0.059	0.083
GDS (score)	20.23 ± 1.23	17.27 ± 1.20	-2.96 (-3.65, -2.26)	20.86 ± 1.55	18.64 ± 1.43	-2.23 (-2.82, -1.63)	0.73 (-1.61, 0.16)	0.105	0.061

Data are expressed as mean ± standard deviation or mean (95% confidence interval)^a^

^*^Significant time and group interaction

T × G, time and group interaction, *MMSE *Mini-mental state examination, *POMS *Profile of mood states, *GDS *Geriatric depression scale

Partial *η*^2^: effect size (small, 0.01; medium, 0.06; large, 0.14)

#### Mood state

Table 3 shows the mood states according to time between the experimental and control groups (Table 3) (Fig. 3). There were no significant differences between the groups with respect to time (F = 3.781, *df* = 1, *p* = 0.059). The mood state scores of the experimental and control groups significantly decreased after the intervention (*p* < 0.001 for both).

#### Depression state

Table 3 shows the depression states of the experimental and control groups (Table 3) (Fig. 3). There was no significant interaction between time and group on the GDS (F = 2.751, *df* = 1, *p* = 0.105). The depression score of the experimental group significantly decreased after the intervention (*p* < 0.001), and the degree of depression in the control group showed a significant change after the intervention (*p* < 0.001).

#### Functional fitness

Table 4 shows the results of significant changes in body function over time between the experimental and control groups to which the single task program was applied (Table 4) (Fig. 3). There was no significant difference between time and group in terms of physical function (back scratch, F < 0.001, *df* = 1, *p* = 0.986; chair sit and reach, F = 0.001, *df* = 1, *p* = 0.977; 244 cm up and go, F = 1.297, *df* = 1, *p* = 0.261; 2-min step walk, F = 0.170, *df* = 1, *p* = 0.682; 30-sec chair stand, F = 2.528, *df* = 1, *p* = 0.119; left grip strength, F = 0.128, *df* = 1, *p* = 0.722; right grip strength, F = 1.408, *df* = 1, *p* = 0.242; one-leg standing test F = 0.217, *df* = 1, *p* = 0.644). In both groups, significant differences were observed before and after the intervention in all sub-variables assessed by the senior fitness test (*p* < 0.001).

**Table 4:** Functional fitness and activities of daily living

	Dual-task resistance exercise			Resistance exercise			Between-group difference in change scores^a^	* p*-value (T×G)	Partial η^2^
	pre	post	Within-group change score^a^	pre	post	Within-group change score^a^			
Senior fitness test									
Back scratch (cm)	-10.41 ± 11.51	-8.59 ± 11.26	1.82 (1.44, 2.19)	-5.23 ± 9.03	-3.41 ± 8.90	1.82 (1.43, 2.21)	-0.00 (-0.53, 0.52)	0.986	< 0.001
Chair sit & reach (cm)	-8.86 ± 9.76	-6.89 ± 9.35	1.97 (1.10, 2.84)	-5.23 ± 10.85	-3.24 ± 10.71	1.99 (1.58, 2.39)	-0.01 (-0.94, 0.92)	0.977	< 0.001
244 cm up & go (sec)	15.85 ± 4.83	13.40 ± 3.71	-2.45 (-3.52, -1.38)	18.49 ± 5.37	16.65 ± 5.55	-1.85 (-2.12, -1.58)	0.60 (-1.68, 0.47)	0.261	0.030
2-min step Walk (number)	26.91 ± 5.33	29.40 ± 5.41	2.50 (1.99, 3.01)	27.18 ± 4.61	29.82 ± 4.66	2.64 (2.17, 3.10)	-0.14 (-0.80, 0.53)	0.682	0.004
30-sec chair stand (number)	7.41 ± 1.92	9.59 ± 1.74	2.18 (1.59, 2.77)	7.77 ± 2.16	9.41 ± 2.46	1.64 (1.24, 2.04)	0.55 (-0.15, 1.24)	0.119	0.057
Grip strength - Lt. (kg)	15.70 ± 7.34	17.19 ± 7.12	1.50 (0.99, 2.00)	17.42 ± 7.97	18.81 ± 7.88	1.39 (1.05, 1.73)	0.10 (-0.49, 0.70)	0.722	0.003
Grip strength - Rt. (kg)	16.97 ± 6.86	19.11 ± 7.16	2.13 (1.68, 2.58)	18.46 ± 8.58	20.96 ± 8.66	2.50 (2.04, 2.96)	-0.37 (-0.99, 0.26)	0.242	0.032
One leg standing (sec)	3.10 ± 0.83	3.57 ± 0.92	0.48 (0.35, 0.60)	2.71 ± 0.58	3.15 ± 0.56	0.45 (0.38, 0.52)	0.03 (-0.11, 0.17)	0.644	0.005
ADL (score)	13.41 ± 1.18	10.86 ± 0.94	-2.55 (-3.09, -2.00)	13.82 ± 1.33	11.36 ± 1.00	-2.45 (-3.08, -1.83)	0.09 (-071, 0.89)	0.820	0.001

Data are expressed as mean ± standard deviation or mean (95% confidence interval)^a^

*EG *Experimental group, *CG *Control group, T × G, time and group interaction, *T *Time, *G *Group, *SFT *Senior fitness test, *ADL *Activities daily of living

Partial *η*^2^: effect size (small, 0.01; medium, 0.06; large, 0.14)

#### Activities of daily living

Table 4 shows the changes in ADL over time between the experimental group to which the cognitive management exercise program was applied and the control group to which the single-task exercise program was applied (Table 4) (Fig. 3). There was no significant difference between the groups with respect to time spent in ADL (F = 0.052, *df* = 1, *p* = 0.820). The ADL score of the experimental group significantly improved after the intervention (*p* < 0.001), and the ADL score of the control group significantly changed after the intervention (*p* < 0.001).

## Discussion

This study investigated the effects of dual-task resistance exercise on cognitive function, mood state, depressive state, functional fitness, and ADL in older adults with cognitive impairment. Compared to the resistance exercise group, there was a significant improvement in cognition in the dual-task resistance exercise group. Both dual-task and resistance exercises significantly improved mood, depression, functional fitness, and ADL.

The importance of physical activity is gradually gaining attention, as previous studies have shown that an increase in physical activity improves cognitive function in older adults [44]. However, the debate continues how much more cognitive improvement benefits dual-task exercises have compared to single-task exercises [45]. The results of this study showed that performing cognitive tasks had a significant advantage in improving cognitive function when the same amount of exercise was performed. This can be interpreted in the same context as recently published studies. In various participants, dual-tasks have been found to have advantages in cognitive function compared with single tasks [45–49]. These results suggest that secondary components, such as inhibition, planning, and execution of motor response, which activate executive functions required to respond to external stimuli, can provide better cognitive benefits when they work together with exercise [50].

Even in the active control group that performed a resistance exercise, cognitive function showed improvement compared to that before the intervention. A previous study have also confirmed Improvements in cognition when resistance exercise was performed in older adults with reduced physical activity [51]. Improvements in cognitive function were confirmed in both groups; however, there was a significant difference in the time-group interaction between the experimental and control groups, indicating that the health benefits of the dual-task were more effective when the intervention was performed for the same period. The human body routinely performs multiple tasks simultaneously, a concept known as dual-tasking, which is crucial for daily functioning. Older adults, especially those experiencing declines in cognitive and physical capabilities, often struggle with these simultaneous demands. Decreased cognitive and physical activity can lead to further declines in overall function [52, 53]. The results of this study showed that managing cognitive and physical functions in older adults through exercise is feasible.

Resistance exercise not only has a positive effect on cognitive function [51], reduces depression and anxiety [54, 55], and increases self-esteem and psychological well-being [56, 57]. Similar to these previous studies, both the dual-task and resistance exercise groups showed improvements in mood and depression before and after the intervention. This is not different from the results that the intensification of depression was highly correlated with cognitive functions, such as impairment of executive function and processing speed [58], and that an increase in physical activity had a positive effect on improving mood and depression in older adults [59, 60]. According to a report by Blumenthal et al., when older adults with depression were asked to exercise regularly, the depression and recurrence rates were lower in the exercise group than in the group taking antidepressant drugs for the same period [61]. Combining this with the results of this study, it is suggested that regular exercise can be as effective as drug treatment in improving depression in older adults. Additionally, physical activity, as facilitated through group exercise, such as the intervention used in this study, appears to have strengthened social networks and provided psychological stability [62–64].

Older adults who engage in regular physical activity are less likely to experience reduced mobility, perform better in ADLs and have higher levels of functional fitness [65]. Furthermore, compared to the elderly population with no physical activity at all, those engaging in physical activity have a lower risk of developing functional limitations, can maintain independence, improves quality of life, and significantly reduce the risk of death [66]. For older individuals with cognitive impairment, engaging in appropriate physical activity becomes challenging, which can make cognitive impairment more severe [67, 68]. In our study, functional fitness and ADL in both groups significantly improved after the intervention. It is presumed that participants who had low physical activity at a nursing institution experienced increased physical strength and improved efficiency in ADL by continuously practicing regular physical activity [69]. This means that social medical expenses can be reduced by preventing the aggravation of disabilities and diseases experienced in daily life through exercise [70].

Although the importance of physical functioning is greatly emphasized across all age groups, maintaining and improving cognitive function tends to be seen as a limited social problem in some populations [71]. However, as mentioned earlier, cognitive function is closely related to depression and has a social advantage in that it increases the quality of life of older adults by inducing the resolution of negative emotions through the improvement of cognitive function. Therefore, the maintenance and improvement of cognitive function in human health promotion is an issue that should be considered along with physical function [72].

This study presents a specific approach to exercise interventions for older adults with cognitive impairment by comparing the effects of dual-task resistance exercise against resistance training. Dual-task resistance exercise demonstrated greater effectiveness in improving cognition while producing equivalent results to resistance exercise applied at the same frequency and duration. This study contributes to the understanding of exercise science by providing a comprehensive assessment of how such interventions impact cognition, mood, depression, physical function, and activities of daily living. The findings suggest dual-task resistance exercises may serve as a more effective method for improving cognitive and physical health in aging populations. Additionally, it contributes to the growing body of evidence supporting dual-task exercise programs as essential tools in managing cognitive health. Lastly, the study lays a foundation for future research, encouraging further exploration into optimizing exercise interventions for cognitive improvement among older adults.

This study has some limitations. First, because the participants knew the group to which they belonged, the possibility of the risk of performance bias cannot be excluded. Second, by comparing only before and after the intervention, it was not possible to determine the most appropriate intervention dose. Third, since follow-up assessments were not performed, the persistence of the intervention effect cannot be determined. Lastly, MMSE used in cognitive assessment is a tool with high reliability and validity, but individual characteristics such as education level or social background may influence the results, and that it may have limitations in detecting cognitive impairment at an early stage or certain types of cognitive impairment [73].

## Conclusion

The 6-week dual-task resistance exercise program was more effective than the resistant exercise program in improving cognitive function in older adults with cognitive impairment. Both dual-task resistance exercise and resistance exercise groups significantly improved mood, depression, functional fitness, and ADL after the intervention. For mood, depression, functional fitness, and ADL, equivalent effects were confirmed between dual-task resistance exercise and resistance exercise groups. Based on this study’s results, we propose using dual-task resistance exercises for cognitive and physical health management in the older adult population.

## Data Availability

The datasets used and/or analyzed during the current study are available from the corresponding author on reasonable request.

## Abbreviations

ADL: Activities of Daily Living
GDS: Geriatric Depression Scale
MMSE: Mini-Mental State Examination
POMS: Profile Of Mood States
SFT: Senior Fitness Test
